# Laser Photobiomodulation: What Are the Ideal Parameters for Each Type of Laser Used in Dental Practice to Promote Fibroblast Proliferation and Differentiation? A Systematic Review

**DOI:** 10.3390/life15060853

**Published:** 2025-05-26

**Authors:** Roberta Iaria, Paolo Vescovi, Pierpaolo De Francesco, Ilaria Giovannacci

**Affiliations:** 1 Oral Medicine and Oral Surgery Laser Unit, University Centre of Dentistry, Department of Medicine and Surgery, University of Parma, 43125 Parma, Italy; paolo.vescovi@unipr.it (P.V.); pierpaolo.defrancesco@unipr.it (P.D.F.); ilaria.giovannacci@gmail.com (I.G.); 2 PhD Program in Molecular Medicine (XXXIX Cycle), University of Parma, 43125 Parma, Italy

**Keywords:** laser photobiomodulation, irradiation parameters, fibroblast proliferation, fibroblast differentiation, in vitro studies

## Abstract

Laser photobiomodulation (LPMB) is a non-invasive therapy that relies on the use of low-power lasers. The literature supports the positive effect of LPBM on tissue regeneration, since it reduces the timing of the inflammatory phase, promoting the proliferative phase of the process. From a purely clinical point of view, the breadth of lasers currently available for dental use makes it difficult to identify unambiguous parameters that can guarantee the best regenerative effect. Therefore, the aim of the present systematic review is to identify the best irradiation parameters for each type of dental laser that can provide the best effects on fibroblast proliferation and differentiation. The literature was searched through the following electronic databases: Medline, Scopus, Web of Science, Springer, and Cochrane, while respecting the PRISMA 2020 guidelines. In vitro studies conducted on human fibroblast cells, drafted in English between 2004 and 2025, were included. A total of 17 papers assessing the effects of diode lasers at different wavelengths (445 nm, 635 nm, 650 nm, 660 nm, 670 nm, 685 nm, 810 nm, 830 nm, 915 nm, 940 nm), CO2 lasers (10.6 µm), and Er:YAG lasers (2940 nm) were included. What can be concluded from the present review is that, for the same wavelength, the wide variability in the results obtained from each study makes it complicated to identify unambiguous parameters for each laser device that can guarantee the best effect on fibroblast proliferation and differentiation.

## 1. Introduction

Laser photobiomodulation (LPBM) is a non-invasive therapy involving the use of low-power lasers. In 1973 Mester et al. first demonstrated that the interaction of laser light with tissues generates photochemical processes resulting in beneficial effects, accelerating the wound healing process and reducing pain [1]. Lasers used for photobiomodulation fall into class III, which are lasers with an emitted power level of less than 500 mW. In odontostomatology clinical practice, laser photobiomodulation is employed for the management of various oral diseases. To date, evidence of beneficial effects has been described for the treatment of oral lichen planus (OLP) [2,3], oral mucositis [4], and oral ulcerative lesions, like recurrent aphthous stomatitis (RAS) [5]. Moreover, it seems to be effective as a supportive therapy for oral pemphigus vulgaris [6] and for medication-related osteonecrosis of the jaws.

The papers currently available in the literature show that laser light targets are represented by chromophores located inside mitochondria. As concluded by Kuna in 1999, due to its four redox active metal centres (the binuclear CuA, CuB, heme a, and heme a3), cytochrome c oxidase (CCO) represents the main photo acceptor, sensitive to monochromatic visible light and near-infrared (NIR) light [7]. Cytochrome c oxidase stimulation by wavelengths ranging between 600–810 nm results in an increase in mitochondrial activity and, thus, in the enhanced production of ions and molecules such as nitric oxide (NO), ATP, calcium ions, and reactive oxygen species (ROS) [8]. Modifications linked to the stimulation of CCO lead to an increase in cell proliferation and differentiation and the activation of transcription factors. More specifically, moderate concentrations of reactive oxygen species (ROS) play a crucial role in several stages of differentiation, including lineage specification and cell fate decisions [9]. Positive effects are reported for low concentrations of ROS; higher concentrations of these species have harmful effects on cells. In order to minimize cytotoxic effects, the regulation of ROS concentration is ensured by negative feedback mechanisms [10]. Longer wavelengths seem to be activated by different targets, namely the transient receptor potential (TRP) family, which is sensitive to radiations from 980 nm to 1064 nm [10].

To date, different kinds of lasers are available for dentistry practice.

The aim of the following systematic review is to assess the best irradiation parameters for each kind of laser in order to achieve the best effect on fibroblast proliferation and differentiation.

## 2. Materials and Methods

### 2.1. Main Question

Our systematic review was conducted with the aim of answering the following main question: for each type of laser used in dentistry, which parameters provide the best effects on fibroblast proliferation and differentiation?

### 2.2. PICO Statement

“In order to assess the effect of laser photobiomodulation on human fibroblast (Population) differentiation and proliferation, we conducted a systematic review of in vitro studies evaluating the effectiveness of different laser wavelengths (Intervention) compared to no treatment (Comparison) in promoting their proliferation and differentiation (Outcome)”.

### 2.3. Search Strategy

The available literature was searched using the main digital databases, including Medline (1997), Web of Science (1956), Scopus (2004), and the Cochrane database (1993), by selecting a time range from 2004 to 2025. In order to screen those articles that could answer the main question, the following keywords were used: “fibroblasts differentiation AND photobiomodulation”, “fibroblasts proliferation AND photobiomodulation”.

### 2.4. Study Selection

Our research was conducted following the Preferred Reporting Items for Systematic Reviews and Meta-Analysis (PRISMA) statement published in 2009 [11].

**Scheme 1:**
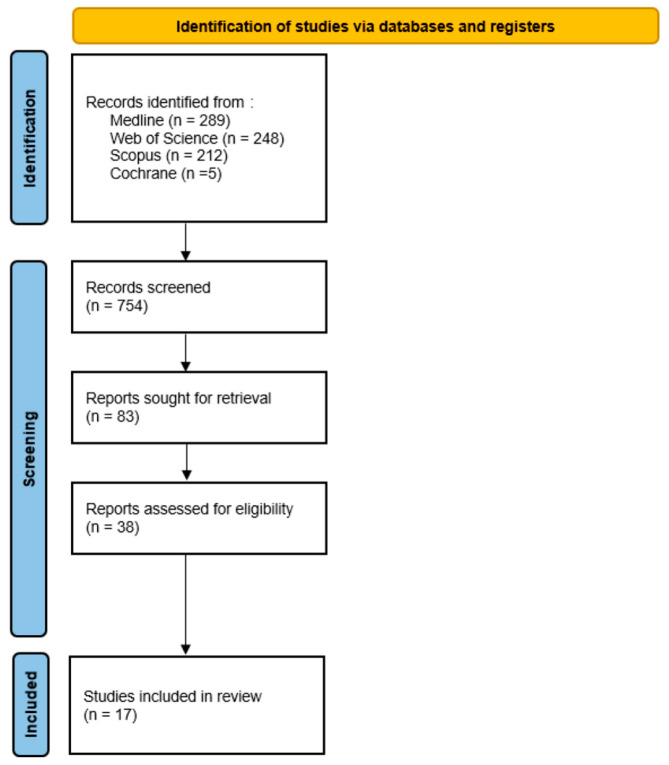
A flowchart of the study according to the 2020 PRISMA checklist.

The selection of articles for inclusion was conducted by two reviewers and required strict screening steps: after the initial shortlisting of articles according to keywords, time range, and item type, papers were selected according to title. From reading the abstracts of the screened articles, the studies that met the inclusion criteria were chosen. Finally, the full text of each article was read and assessed for inclusion in the final selection (Scheme 1).

### 2.5. Eligibility Criteria

*Inclusion criteria*

1. In vitro studies evaluating the best settings to promote proliferation and differentiation with lasers used in dentistry.
2. Studies conducted in vitro exclusively on human fibroblast cells.
3. Systematic reviews and metanalyses, or narrative reviews.

*Exclusion criteria*

1. In vitro studies conducted on cells other than fibroblasts.
2. In vivo studies.
3. Papers in languages other than English.
4. Clinical cases or clinical series, reviews, opinion articles, trials conducted on animals, letters from editors, book chapters, appendices, bibliographies, indexes, and articles whose full text is not available.
5. Studies that used very high fluence, with a density greater than 500 J/cm^2^.
6. Articles that did not mention power or fluence rate.

### 2.6. Level of Evidence

In order to assess the methodological quality of each study included in our systematic review, we decided to use the item bank proposed by Whaley et al. in 2024 [12], which is specifically aimed at in vitro studies.

For all studies, the criteria given in the checklist in Table 1 were evaluated and those that were satisfied were marked with an ‘x’.

## 3. Results

### 3.1. Cell Culture

All selected articles consist of studies performed in vitro on human cell cultures. Specifically, ten of the seventeen articles were conducted on gingival fibroblast cells [14,15,16,17,18,19,20,22,23,24], two of them were conducted on dermal fibroblast cells [21,30], one on human Caucasian foetal foreskin fibroblasts [13], one on foetal human skin fibroblast cells (ATCC CCD1070SK) [25], and one on the human epithelial fibroblast CCD-1064Sk [26].

### 3.2. Laser Wavelength

The selection of articles in the literature was conducted by choosing trials carried out with laser devices employed in clinical dental practice.

#### 3.2.1. Diode Lasers

Of the articles included in the review, twelve employed a diode laser; of these, three evaluated the cellular effect of 980 nm wavelength lasers [14,18,25], two articles reported the action of 940 nm lasers [15,26], and two reported the action of 810 nm lasers [15,16]. The remaining five articles reported the action of diode lasers at different wavelengths: 445 nm [17], 635 nm [18], 650 nm [30], 660 nm [20], 670 nm [13], 685 nm [22], 830 nm [22], 915 nm [23]. Most of the included studies entailed the application of lasers in continuous mode, while two studies, one with a 980 nm diode laser [14] and one with a 2940 nm erbium YAG laser [24], investigated the effect of irradiation in pulsed mode (Table 2).

Tripodi et al. studied the biological effects of laser photobiomodulation in the polarised mode, reporting an increase in cell proliferation and a cytoprotective effect when using a 670 nm diode laser with a fluence of 1 J/cm^2^ in the above-mentioned mode [13].

In the investigation conducted by Khalaj et al. in 2023, the evaluation of the effects of a 980 nm diode laser used in both pulsed and continuous mode revealed that both modalities had positive effects on the proliferation of human gingival fibroblasts. Namely, they observed that 24 h after laser exposure, the group receiving continuous irradiation with an energy density of 5.2 J/cm^2^ showed the highest proliferation rate, while after 48 and 72 h, the pulsed mode group, irradiated with a frequency of 50 Hz and an average energy density of 5 J/cm^2^, showed a significant increase in proliferation [14].

In 2020, Ladiz et al. published the results of a study evaluating the effect of a single session of 810 nm and 940 nm diode lasers used alone and in combination. They concluded that the 810 nm laser at 0.5 J/cm^2^ showed a higher proliferation rate at 24 and 72 h, while the best effect on proliferation after 72 h was achieved with an energy density of 1.5 J/cm^2^. For the 940 nm group, the best proliferation rate was reached at an energy density of 2.5 J/cm^2^. The combined use of these wavelengths showed better results [15].

In their in vitro study, Karoussis et al. evaluated the effects of different energy densities (2, 4, 6, and 12 J/cm^2^) of photobiomodulation using an 810 nm diode laser. From their observation, the most advantageous energy density for improving gingival fibroblast proliferation for up to 72 h was 12 J/cm^2^ [16].

Etmadi et al. studied the effect of a 445 nm laser on the proliferation and migration of cultured human gingival fibroblast cells. They evaluated three different power levels: 200 mW (irradiation times of 5, 10, 15, and 20 s); 300 mW (irradiation times of 5, 10, and 15 s); and 400 mW (irradiation times of 5 and 10 s). The results revealed that laser irradiation at a power density of 400 mW/cm^2^ with irradiation times of 10 and 15 s (4 and 6 J/cm^2^) exerted the best statistically significant effect on cell viability and proliferation [17].

In another study conducted in 2021, Etmadi et al. compared the effects of different energy densities and diode laser wavelengths on HGF proliferation. Specifically, the proliferation rate of cells irradiated with wavelengths of 635, 660, 808, and 980 nm and densities of 1, 1.5, 2.5, and 4 J/cm^2^ was evaluated after 1, 3, and 5 days using the MTT assay. The study concluded that, to achieve the highest rate of cell proliferation, a 980 nm wavelength with energy densities of 1, 1.5, and 4 J/cm^2^ and a 635 nm wavelength with an energy density of 4 J/cm^2^ are the most desirable laser radiation settings [18].

Kimia Hafezi Motlagh et al. studied the effect of two different wavelengths, 445 nm with a power level of 50 mW and a dose of 4 J/cm^2^ and 660 nm with a power level of 50 mW and a dose of 4 J/cm^2^, both used in continuous mode. They irradiated a culture of human gingival fibroblasts six times, with a time interval of 48 h, at a cross-sectional area of 1 cm^2^. The results of the study at day 7 and day 14 showed the positive effect of the low-power 660 nm laser on fibroblast proliferation compared to the 445 nm laser. In fact, the cell proliferation rates at days 7 and 14 were lower in the group subjected to 445 nm laser irradiation than in the other groups, including the non-irradiated control group [20].

In their study conducted on human gingival fibroblasts, Isil Saygun et al. analysed the effect of a 685 nm wavelength laser using an output power level of 25 mW. Cells were exposed to laser light for a period of 140 s, with the total amount of energy applied being 2 J/cm^2^. Specifically, the samples were divided equally into three groups: the first received a single dose of irradiation; in the second group the same dose was applied for two consecutive days; and the last, the control group, was not irradiated. The study results showed a more pronounced increase in cell proliferation in both laser groups than in the control group [22].

Saeed Sadatmansouri et al. designed a study to evaluate the effect of a 915 nm diode laser on the vitality and viability of human gingival fibroblasts. Specifically, they evaluated four energy densities: 1, 2, 3, and 4 J/cm^2^. A significant increase in cell viability and vital capacity was recorded at an energy density of 3 J/cm^2^ compared to the control group (*p* = 0.007) and at the irradiation dose of 1, 2 J/cm^2^ on day three (*p* < 0.05). After five days, a significant increase in energy density of 2, 3, and 4 J/cm^2^ compared to 1 J/cm^2^ was detected [23].

In 2008, Skopin et al. evaluated the biological effect of a 980 nm diode laser at different doses of energy density on foetal human skin fibroblast cells using a cellular wound healing model. Specifically, they conducted two experiments: the first compared different exposure intensities with the same exposure duration, and the second compared different exposure durations with the same exposure intensity. For the first experiment, energy densities ranged from 3.1 to 14.4 J/cm^2^ (26–120 mW/cm^2^ for a 2 min exposure). For the second experiment, a power level of 73 mW/cm^2^ was used and exposure durations varied from 20 s to 15 min, with exposure doses ranging from 1.5 to 66 J/cm^2^. The study concluded that significant increases in cell growth are achievable with a 2 min exposure period at 26–73 mW/cm^2^ (Po0.01) and 97 mW/cm^2^ (Po0.05). Increased levels of cell proliferation were described with a power level of 73 mW/cm^2^ and a duration of between 50 s and 2 min (8.8–21.9 J/cm^2^). In contrast, a long exposure period of 15 min (65.7 J/cm^2^) had no positive effect on cell growth, again suggesting that excessive exposure to laser light may negate the benefits of lower exposure [25].

The paper by Rebeca Illescas-Montes et al. demonstrates that in vitro treatment of human epithelial fibroblasts with a 940 nm diode laser modulates the expression of the human fibroblast markers FGF, CTGF, VEGF, TGF-β1, TGFβR1, TGFβR2, TGFβR3, α-actin, fibronectin, decorin, elastin, DDR2, and MMP2, thus promoting fibroblast proliferation, migration, and/or maturation. The study employed a 940 nm laser with a maximum power level of 10 W, a spot diameter of 400 microns, a power level of 0.5 W, and an energy density of 4 J/cm^2^ in continuous mode. The results showed an intense expression of fibronectin and α-actin after treatment of laser-treated fibroblasts. The results of the study support the thesis that laser treatment increases the growth and migration of fibroblasts and induces their differentiation, thus enhancing the wound healing process [26].

Karimi et al. analysed the effects of two energy densities, 2 and 4 J/cm^2^, for three different diode laser wavelengths in continuous irradiation mode (660 nm, 808 nm, and 915 nm) on HGF proliferation [27]. They concluded that the highest proliferation rate was reached using a 915 nm laser with an energy density of 4 J/cm^2^: the best effect of this wavelength compared to the other groups was observed at both time points (one and three days after irradiation). Moreover, among the two 915 nm groups, 4 J/cm^2^ exhibited better effects compared to 2 J/cm^2^ [27].

Oyebode et al. studied the proliferation rate induced by irradiation from a 830 nm laser with 5 J/cm^2^ on different fibroblast cell models: normal (N) unstressed cells; normal wounded (NW); diabetic (D); diabetic wounded (DW); and hypoxic diabetic wounded (HDW) [28]. The results obtained from the cell proliferation analysis conducted with the BD Pharmingen™ BrdU FITC Flow Kit showed that irradiation stimulated cells to enter and initiate the S and G2/M phases from the G0/G1 phase [28].

The last study was conducted using a HeNe laser with a wavelength of 632.8 nm [29]. In this study Hawkins and Abrahamse assessed the impact of laser irradiation on human skin fibroblast proliferation by administering a single dose of 0.5, 2.5, 5, 10, or 16 J/cm^2^ on two consecutive days. They concluded that an enhancement of proliferation, with the complete absence of adverse effects on the cells’ structure, can be achieved with an energy density of 5 J/cm^2^, while higher doses such as 10 or 16 J/cm^2^ appear to be correlated with an increase in cytotoxicity and DNA damage.

#### 3.2.2. Erbium YAG Laser

Two of the selected articles investigate the effect of photobiomodulation on fibroblast cells using Er:YAG lasers (2940 nm) [19,24].

The article published in 2015 by Ogita et al. reports the results of an in vitro study conducted with the aim of determining the proliferation and demolition rates of a culture of human gingival fibroblasts exposed to Er:YAG laser photobiomodulation. Three output energy levels were investigated: 30, 40, and 50 mJ/pulse with an irradiation time of 30 s, which corresponded to an energy density of 1.84, 2.35, and 2.90 mJ/cm^2^ per pulse. The results report that laser irradiation at 2.11 and/or 2.61 J/cm^2^ significantly increased cell proliferation by 1.13 to 1.22 times (*p* < 0.05) in six independent experiments using HGF from two different subjects [19].

In a study conducted in 2005 by Pourzarandian et al., the effectiveness of different energy densities (1.68, 2.35, 3.37, or 5.0 J/cm^2^) of an Er:YAG laser used in pulsed mode (20 Hw) on the proliferation of human gingival cells was explored. That study states that the optimal energy density to stimulate cel growth was 3.37 J/cm^2^. They also demonstrated that higher energy densities are correlated with a decrease in cell proliferation and an increase in lactate dehydrogenase levels [24].

#### 3.2.3. CO_2_ Laser

Lastly, one of the eighteen studies was conducted with a CO_2_ laser (10.6 µm) [21]. In that study Shingyochi et al. investigated the effects of CO_2_ laser photobiomodulation on dermal fibroblast proliferation and migration. The laser system was used in the continuous wave mode and with different irradiation power levels: 0.1 J/cm^2^ (52.08 mW/cm^2^, 2 s), 0,5 J/cm^2^ (52.08 mW/cm^2^, 10 s), 1.0 J/cm^2^ (52.08 mW/cm^2^, 20 s), 2.0 J/cm^2^ (52.08 mW/cm^2^, 40 s), and 5.0 J/cm^2^ (520.83 mW/cm^2^, 10 s). The results showed that CO_2_ laser photobiomodulation at 1.0 J/cm enhanced fibroblast proliferation. Similar results were obtained for fibroblast migration. Thus, it is clear from this experiment that CO_2_ laser irradiation at 1.0 J/cm is the most effective for fibroblast activation [21].

## 4. Discussion

The wound healing process is a complex and fascinating chapter of our physiology involving a highly co-ordinated succession of overlapping phases and the specific action of different cell species. Notably, the cascade of wound healing is divided into four phases that occur in the following chronological order: haemostasis, inflammation, proliferation, and remodelling [31,32]. The haemostasis phase begins immediately after the injury and involves the recall of platelets at the wound site due to the effect of the von Willebrand factor. During this phase, platelet adhesion and activation occurs, resulting in the release of secretory granules and the recruitment of further platelets. The aggregation of these cell fragments results in platelet plug formation, thus interrupting the outflow of blood and setting the basis for the temporary matrix formation. Once the first layer of platelets forms at the vascular injury site, the recruitment of more platelets to the growing thrombus relies on the formation of fibrinogen bridges [33]. Inflammation is a necessary phase of the wound healing process that is mainly aimed at cleansing wounds of infection and removing cell debris [34]. Damage-associated molecular patterns (DAMPs), hydrogen peroxide (H_2_O_2_), lipid mediators, and chemokines released by injured cells also provide signals for the recruitment of inflammatory cells, especially neutrophils [35]. The proliferative phase represents the most crucial stage of the wound healing process, since alterations of this step can lead to unfavourable outcomes. Indeed, a prolonged inflammatory phase or an excess of cell proliferation could lead, respectively, to the persistence of the defect (chronic wound) or an excess of scar tissue, consisting mainly of the formation of hypertrophic or keloid scars [36]. The cell species that seem to play a decisive role in the proliferative phase are fibroblasts [37]. Their migration to the wound site is promoted by several cytokines, including transforming growth factor beta (TGF-β), platelet-derived growth factor (PDGF), epidermal growth factor (EGF), fibroblast growth factor (FGF), tumour necrosis factor alpha (TNFα), and interleukin 1 (IL-1) (citazione). Once at the site, the fibroblasts begin to synthesise and secrete metalloproteinases (MMPs), protease enzymes involved in the provisional matrix degradation. During this phase, fibroblasts undergo phenotypic changes that result in increased contraction and production of collagen and fibronectin, the main components of the final extracellular matrix. Concomitantly, these changes lead to a decrease in cell proliferation [38]. The progressive accumulation of collagen gradually leads to the conversion of the newly formed granulation tissue into a scar. The remodelling phase involves establishing a balance between the formation and degradation of the extracellular matrix, orchestrated by growth factors, in particular, PDGF, EGF, IL-1, and TGF-β.

Photobiomodulation is a non-invasive and painless therapy involving the use of laser devices with wavelengths in the red and near-infrared ranges, i.e., between 600 and 1000 nm, and power levels ranging from 5 to 500 mW. The available literature confirms the analgesic effect that lasers exert on inflammation-related pain by lowering the levels of prostaglandin E2 (PGE2), interleukin-1 beta (IL-1 beta), tumour necrosis factor-alpha (TNF-alpha), cellular influx of neutrophils and granulocytes, oxidative stress, oedema, and bleeding in a in a way that is proportional to the dose administered [39]. Furthermore, as highlighted by Jere et al., laser photobiomodulation modulates fibroblast cellular autocrine signalling, particularly the EGF/EGFR loop, by activating the JAK/STAT signalling pathway and increasing cellular proliferation and migration [40].

Consequently, in the wound context, the application of low-power lasers not only performs a disinfectant action [41], but also exerts healing-promoting effects by limiting the timing of the inflammation phase and inducing the next phase of cell proliferation. The studies selected for this review investigate the proliferative and differentiative effects of the various energy densities of different laser wavelengths on human fibroblast cells.

Something that stands out from the review conducted is that, to date, it is not possible to identify unambiguous parameters for each type of laser due to the variability in the conduction of the various studies and the limitations of the comparability of the available papers. What has been observed is that, in the great majority of cases, for the same cell culture and type of laser, the parameters indicated as ‘ideal’ for achieving an enhancing effect on cell proliferation and differentiation differ from one study to another, making the identification of specific values challenging.

The previous observation can be confirmed by analysing the two studies conducted on human gingival fibroblast cells irradiated with diode lasers at a wavelength of 810 nm. In the study conducted by Karoussis et al. [16], the energy density capable of increasing cell proliferation is 12 J/cm^2^, a very high value compared to that identified in the study by Ladiz et al. [15], which was between 0.5 J/cm^2^ (at 24 and 72 h after irradiation) and 1.5 J/cm^2^ (72 h after treatment). Wide energy density ranges were also obtained from the comparison of studies using 940 nm wavelength diode lasers: the results of the in vitro study conducted by Rebeca Illescas-Montes et al. [26] report an increase in fibroblast growth, migration, and differentiation at an energy density of 4 J/cm^2^, while the study by Ladiz et al. [15] shows positive effects on cell proliferation at 2.5 J/cm^2^.

Less significant differences were found between the studies conducted on human gingival fibroblast cells with a 980 nm diode laser: according to Khalaj et al. [14], the highest proliferation rate possible when using the continuous irradiation mode is obtained with an energy density of 5.1 J/cm2, while the in vitro study conducted in 2021 by Etmadi et al. [18] identified 1, 1.5, and 4 J/cm^2^ as the energy densities associated with the best proliferative effect.

In contrast, the studies performed using 660 nm wavelength diode lasers provided similar results, suggesting an optimal energy density of 4 J/cm^2^ [20]. The same value was also identified as promoting fibroblast proliferation in studies using diode lasers with a wavelength of 915 nm [23].

In conclusion, given the high variability of the data currently available in the literature concerning the laser device parameters that can provide the best effect on human fibroblast proliferation and differentiation, it is necessary to carry out studies that are as standardised as possible for each wavelength.

Lastly, the authors consider it crucial to specify that the parameters identified in this review refer to those to which fibroblasts respond positively when lasers are applied to cell cultures; but, taking into account the fact that, in the clinical field, light interacts with complex anatomical structures, it is not certain that the values identified would obtain the same response in vivo. So, it would be interesting to conduct further investigations to validate their applicability in tissues.

## Figures and Tables

**Table 1:** The item bank for the methodological quality assessment of in vitro studies.

	Nicholas Tripodi et al. 2022 [13]	Saina Khalaj et al. 2023 [14]	Mohammd Ayoub Rigi Ladiz, et al. 2020 [15]	Ioannis K. Karoussis et al. 2021 [16]	Ardavan Etemadi et al. 2020 [17]	Ardavan Etemadi et al. 2021 [18]	Mayumi Ogita et al. 2014 [19]	Kimia Hafezi Motlagh et al. 2023 [20]	Yoshiaki Shingyochi et al. 2017 [21]	Isil Saygun et al. 2007 [22]	Saeed Sadatmansouri and et al. 2022 [23]	Amir Pourzarandian et al. 2005 [24]	Mark D. Skopin and Scott C. Molitor 2008 [25]	Rebeca illescas-Montes et al. 2019 [26]	Karimi et al. 2024 [27]	Oyebode et al. 2022 [28]	Hawkins and Abrahamse 2006 [29]
	Tools Published as Stand-Alone Manuscripts (n = 17)																
**1 Objective**	**x**	**x**	**x**	**x**	**x**	**x**	**x**	**x**	**x**	**x**	**x**	**x**	**x**	**x**	**x**	**x**	**x**
**1.1 Hypothesis**	**x**	**x**	**x**	**x**	**x**	**x**	**x**	**x**	**x**	**x**	**x**	**x**	**x**	**x**	**x**	**x**	**x**
**1.1.1 Mode of research**	**x**	**x**	**x**	**x**	**x**	**x**	**x**	**x**	**x**	**x**	**x**	**x**	**x**	**x**	**x**	**x**	**x**
**1.1.2 Theoretical basis**	**x**	**x**	**x**	**x**	**x**	**x**	**x**	**x**	**x**	**x**	**x**	**x**	**x**	**x**	**x**	**x**	**x**
**1.2 Justification**	**x**	**x**	**x**	**x**	**x**	**x**	**x**	**x**	**x**	**x**	**x**	**x**	**x**	**x**	**x**	**x**	**x**
**1.2.1 Importance**																	
**1.2.2 Context**																	
**2 Test or Experimental System**	**x**	**x**	**x**	**x**	**x**	**x**	**x**	**x**	**x**	**x**	**x**	**x**	**x**	**x**	**x**	**x**	**x**
**2.1 Theory by which experiment is test of hypothesis**	**x**	**x**	**x**	**x**	**x**	**x**	**x**	**x**	**x**	**x**	**x**	**x**	**x**	**x**	**x**	**x**	**x**
**2.1.1 PECO characteristics**	**x**	**x**	**x**	**x**	**x**	**x**	**x**	**x**	**x**	**x**	**x**	**x**	**x**	**x**	**x**	**x**	**x**
**2.1.1.1 Population**	**x**	**x**	**x**	**x**	**x**	**x**	**x**	**x**	**x**	**x**	**x**	**x**	**x**	**x**	**x**	**x**	**x**
**2.1.1.2 Exposure or intervention**	**x**	**x**	**x**	**x**	**x**	**x**	**x**	**x**	**x**	**x**	**x**	**x**	**x**	**x**	**x**	**x**	**x**
**2.1.1.3 Comparator or controls**	**x**	**x**	**x**	**x**	**x**	**x**	**x**	**x**	**x**	**x**	**x**	**x**	**x**	**x**	**x**	**x**	**x**
**2.1.1.4 Outcome**	**x**	**x**	**x**	**x**	**x**	**x**	**x**	**x**	**x**	**x**	**x**	**x**	**x**	**x**	**x**	**x**	**x**
**2.1.2 Fit between methods and hypothesis**	**x**	**x**	**x**	**x**	**x**	**x**	**x**	**x**	**x**	**x**	**x**	**x**	**x**	**x**	**x**	**x**	**x**
**2.1.2.1 External validity**																	
**2.1.2.2 Construct validity**																	
**2.1.3 General methodological information**																	
**2.1.3.1 Materials**	**x**	**x**	**x**	**x**	**x**	**x**	**x**	**x**	**x**	**x**	**x**	**x**	**x**	**x**	**x**	**x**	**x**
**2.2 Verification of experimental components**	**x**	**x**	**x**	**x**	**x**	**x**	**x**	**x**	**x**	**x**	**x**	**x**	**x**	**x**	**x**	**x**	**x**
**2.2.1 Authenticity of population, e.g., cell culture**	**x**	**x**	**x**	**x**	**x**	**x**	**x**	**x**	**x**	**x**	**x**	**x**	**x**	**x**	**x**	**x**	**x**
**2.2.2 Exposure**	**x**	**x**	**x**	**x**	**x**	**x**	**x**	**x**	**x**	**x**	**x**	**x**	**x**	**x**	**x**	**x**	**x**
**2.2.3 Outcome occurrence and magnitude**	**x**	**x**	**x**	**x**	**x**	**x**	**x**	**x**	**x**	**x**	**x**	**x**	**x**	**x**	**x**	**x**	**x**
**2.2.4 General validation**	**x**	**x**	**x**	**x**	**x**	**x**	**x**	**x**	**x**	**x**	**x**	**x**	**x**	**x**	**x**	**x**	**x**
**2.2.5 Other materials**																	
**2.3 Good experimental practices**																	
**2.3.1 Controlling for systematic errors**																	
**2.3.1.1 Blinding**																	
**2.3.1.2 Randomisation**																	
**2.3.1.3 Effect modifiers**																	
**2.3.1.4 Allocation concealment**																	
**2.3.1.5 Attrition**																	
**2.3.1.6 Selection**																	
**2.3.1.7 Confounding**																	
**2.3.1.8 Detection**																	
**2.3.2 Controlling for random errors**																	
**2.3.2.1 Replicates**																	
**2.3.2.2 Sample size, e.g., power analysis**																	
**2.3.3 Nonspecific error control**																	
**2.3.3.1 Investigator roles**																	
**2.3.3.2 Adherence to standardised practices**																	
**2.3.4 Conduct according to protocol**																	
**3 Analysis and derivation of findings**																	
**3.1 Generation of results**																	
**3.1.1 Data normalisation and cleansing**																	
**3.1.2 Statistical methods**	**x**	**x**	**x**	**x**	**x**	**x**	**x**	**x**	**x**	**x**	**x**	**x**	**x**	**x**	**x**	**x**	**x**
**3.1.3 Image analysis**													**x**		**x**		
**3.1.4 Visualisation methods**																	
**3.1.5 Complete reporting**																	
**3.1.6 Software**																	
**3.1.7 Raw data, code, etc.**																	
**3.2 Interpretation of results**																	
**3.2.1 Significance**	**x**	**x**	**x**	**x**		**x**	**x**	**x**	**x**	**x**	**x**	**x**	**x**	**x**	**x**	**x**	**x**
**3.2.2 Limitations of experimental approach**					**x**												
**4 Other**																	
**4.1 Interests**																	
**4.2 Summary sections**																	
**4.2.1 Abstract**	**x**	**x**	**x**	**x**	**x**	**x**	**x**	**x**	**x**	**x**	**x**	**x**	**x**	**x**	**x**	**x**	**x**
**4.2.2 Resources**																	
**4.2.3 Captions**																	
**4.3 Ethics**																	
**4.4 Prospects for replication**					**x**												
**Not classifiable under any domain**																	

**Table 2:** An outline of the in vitro studies included in this systematic review.

Author	Year	Type of Paper	Cellular Line	Laser	Wavelength	Output Power/Output Energy	Energy Density (J/cm^2^)	Frequency (Hz)	Irradiation Time (s)	Irradiation Area (cm^2^)	Type of Irradiation	Effect
**Nicholas Tripodi et al.** [13]	2022	Article	Human caucasian foetal foreskin fibroblasts (HFFF2)	Diode	670 nm		1 J/cm^2^	-			Polarized	Enhanced proliferative and cytoprotective effect.
**Saina Khalaj et al.** [14]	2023	Research article	Human gingival fibroblasts	Diode	980 nm		5.2 J/cm^2^	-			Continuous irradiation mode	Higher proliferation rate.
							5 J/cm^2^	50 Hz			Pulsed irradiation mode	Positive effect on cell proliferation.
**Mohammd Ayoub Rigi Ladiz et al.** [15]	2020	Original paper	Human gingival fibroblasts	Diode	810 nm		0.5 J/cm^2^	-			Continuous irradiation mode	Positive effect on cell viability, NO PROLIFERATION.
					810 nm + 940 nm		1.5 J/cm^2^ and 2.5 J/cm^2^	-			Continuous irradiation mode	Higher proliferation rate.
**Ioannis K. Karoussis et al.** [16]	2021	Original paper	Human gingival fibroblasts (hGFs)	Diode	810 nm	500 mW	12 J/cm^2^	-		0.16 cm^2^	Continuous irradiation mode	Increase in cell proliferation rate.
**Ardavan Etemadi et al.** [17]	2020	Original paper	Human gingival fibroblasts	Diode	445 nm	200 and 400 mW	4 to 8 J/cm^2^	-	10 s	0.5 cm^2^	Continuous irradiation mode	Enhanced effect on proliferation and migration.
**Ardavan Etemadi et al.** [18]	2021	Research article	Human gingival fibroblasts (HGF1-PI 1)	Diode	635 nm	220 mW	4 J/cm^2^	-	12 s	0.5 cm^2^	Continuous irradiation mode	Most desirable laser radiation settings to obtain higher proliferation rates.
					980 nm	100 mW	1, 1.5, 4 J/cm^2^	-	2.5, 4, and 10 s	0.5 cm^2^	Continuous irradiation mode	
**Mayumi Ogita et al.** [19]	2014	Original paper	Human gingival fibroblasts	Erbium YAG	2940 nm	1.84, 2.35, and 2.90 mJ/pulse	2.11 and/or 2.61 J/cm^2^	-	30 s		Continuous irradiation mode	Significant enhancement of cell proliferation rate.
**Kimia Hafezi Motlagh et al.** [20]	2023	Method article	Human gingival fibroblasts (HuGu)	Diode	660 nm	50 mW	4 J/cm^2^	-	64 s	1 cm^2^	Continuous irradiation mode	Positive effect on quantity and quality of human gingival fibroblasts.
**Yoshiaki Shingyochi et al.** [21]	2017	Research article	Human dermal fibroblasts (HDFs)	CO_2_	10.6 µm	52.08 mW/cm ^2^	1 J/cm^2^	-	20 s	35 mm	Continuous irradiation mode	Most effective parameter to obtain best effect on proliferation.
**Isil Saygun et al.** [22]	2007	Original paper	Human gingival fibroblasts	Diode	685 nm	25 mW	2 J/cm^2^	-	140 s	1 cm^2^	Continuous irradiation mode	Positive effect on cell proliferation rate with single and double irradiation.
**Saeed Sadatmansouri et al.** [23]	2022	Research article	Human gingival fibroblasts (C-10459 hugu-PI1)	Diode	915 nm		2, 3 and 4 J/cm^2^	-			Continuous irradiation mode	Parameters seem to enhance viability of human gingival fibroblast cells for extended periods.
**Amir Pourzarandian et al.** [24]	2005	Paper	Human gingival fibroblasts (hGF)	Erbium YAG	2940 nm	30 to 350 mJ/pulse	3.37 J/cm^2^	20 Hz		35 mm	Pulsed irradiation mode	Findings showed stimulatory effect of Er:YAG laser on hGF proliferation in laser dose-dependent manner.
**Mark D. Skopin and Scott C. Molitor** [25]	2008	Original paper	Foetal human skin fibroblast cells (ATCC CCD1070SK)	Diode	980 nm	1.5 to 7.5 W 26–120 mW/cm^2^	3.1 to 14.4 J/cm^2^		120 s		Continuous irradiation mode	Increased cell proliferation was obtained with energy density from 8.8 to 21.9 J/cm^2^, while negative effects were observed after long exposure to laser irradiation (65.7 J/cm^2^).
**Rebeca Illescas-Montes et al.** [26]	2019	Scientific report	Human epithelial fibroblast CCD-1064Sk	Diode	940 nm	0.5 W	4 J/cm^2^				Continuous irradiation mode	Both treatments with 940 nm diode laser (single dose or two doses) significantly increased FGF expression, although effect was lower with two doses.
**Karimi et al.** [27]	2024	Original paper	Human gingival fibroblasts (hGF)	Diode	660 nm		2 and 4 J/cm^2^				Continuous irradiation mode	Highest proliferation rate compared to other groups was observed in 915 nm wavelength with energy density of 4 J/cm^2^.
					808 nm							
					915 nm							
**Oyebode et al.** [28]	2022	Original research	Human skin fibroblast cell line	Diode	830 nm		5 J/cm^2^				Continuous irradiation mode	Laser irradiation stimulated cells to enter and initiate S and G2/M phases from G0/G1 phase.
**Hawkins and Abrahamse** [29]	2006	Original paper	Human skin fibroblast (ATCC CRL1502 WS1)	Diode	632.8 nm		0.5, 2.5, 5, 10, 16 J/cm^2^				Continuous irradiation mode	Proliferation and complete absence of adverse effects on cells’ structure can be reached with energy density of 5 J/cm^2^.

## Abbreviations

The following abbreviations are used in this manuscript:

CEBM	Centre for Evidence-Based Medicine
CO_2_	Carbon Dioxide
EGF	Epidermal growth factor
EGFR	Epidermal growth factor receptor
Er:YAG	Erbium: Yttrium-Aluminium-Garnet
HGF	Human gingival fibroblasts
HeNe	Helium-Neon
Hz	Hertz
J/cm^2^	Joule per square centimetre
JAK/STAT	Janus Kinase/Signal Transducer and Activator of Transcription
mJ/cm^2^	Millijoule per square centimetre
mW	Milliwatt
mW/cm^2^	Milliwatt per square centimetre
NO	Nitric oxide
PICO	Patient, Intervention, Comparison, Outcome
PDGF	Platelet-derived growth factor
PRISMA	Preferred Reporting Items for Systematic Reviews and Meta-Analysis
ROS	Reactive oxygen species
TGF-β	Transforming growth factor beta
TRP	Transient receptor potential
µm	Micrometre

## References

[1] Mester E., Korényi-Both A., Spiry T., Scher A., Tisza S. (1973). Stimulation of wound healing by means of laser rays. (Clinical and electron microscopical study). Acta Chir. Acad. Sci. Hung..

[2] Del Vecchio A., Palaia G., Grassotti B., Tenore G., Ciolfi C., Podda G., Impellizzeri A., Mohsen A., Galluccio G., Romeo U. (2021). Effects of laser photobiomodulation in the management of oral lichen planus: A literature review. Clin. Ter..

[3] Nammour S., El Mobadder M., Brugnera A.J., Namour M., Houeis S., Heysselaer D., Vanheusden A., Namour A. (2021). Photobiomodulation Therapy vs. Corticosteroid for the Management of Erosive/Ulcerative and Painful Oral Lichen Planus. Assessment of Success Rate during One-Year Follow-Up: A Retrospective Study. Healthcare.

[4] Cruz A.R., Minicucci E.M., Betini M., Almeida-Lopes L., Neto V.T., Cataneo A.J.M. (2023). Efficacy of photobiomodulation in the treatment of oral mucositis in patients undergoing antineoplastic therapy: Systematic review and meta-analysis. Support. Care Cancer.

[5] AlHerafi E., Hamadah O., Parker S. (2024). Photobiomodulation in recurrent aphthous stomatitis management using three different laser wavelengths. A randomized clinical trial. Lasers Med. Sci..

[6] Amadori F., Bardellini E., Veneri F., Majorana A. (2022). Photobiomodulation laser therapy in pemphigus vulgaris oral lesions: A randomized, double-blind, controlled study. Stomatologija.

[7] Karu T. (1999). Primary and secondary mechanisms of action of visible to near-IR radiation on cells. J. Photochem. Photobiol. B Biol..

[8] Dompe C., Moncrieff L., Matys J., Grzech-Leśniak K., Kocherova I., Bryja A., Bruska M., Dominiak M., Mozdziak P., Skiba T.H.I. (2020). Photobiomodulation-Underlying Mechanism and Clinical Applications. J. Clin. Med..

[9] Palma F.R., Gantner B.N., Sakiyama M.J., Kayzuka C., Shukla S., Lacchini R., Cunniff B., Bonini M.G. (2024). ROS production by mitochondria: Function or dysfunction?. Oncogene.

[10] Zamani A.R.N., Saberianpour S., Geranmayeh M.H., Bani F., Haghighi L., Rahbarghazi R. (2020). Modulatory effect of photobiomodulation on stem cell epigenetic memory: A highlight on differentiation capacity. Lasers Med. Sci..

[11] Moher D., Liberati A., Tetzlaff J., Altman D.G. (2009). Preferred Reporting Items for Systematic Reviews and Meta-Analyses: The PRISMA Statement. PLoS Med..

[12] Whaley P., Blain R.B., Draper D., Rooney A.A., Walker V.R., Wattam S., Wright R., Hooijmans C.R. (2024). Identifying assessment criteria for in vitro studies: A method and item bank. Toxicol. Sci..

[13] Tripodi N., Sidiroglou F., Fraser S., Husaric M., Kiatos D., Apostolopoulos V., Feehan J. (2022). The effects of polarized photobiomodulation on cellular viability, proliferation, mitochondrial membrane potential and apoptosis in human fibroblasts: Potential applications to wound healing. J. Photochem. Photobiol. B Biol..

[14] Khalaj S., Iranpour B., Hodjat M., Azizi A., Kharazifard M.J., Hakimiha N. (2024). Photobiomodulation effects of pulsed and continuous wave near-infrared laser on the proliferation and migration of human gingival fibroblasts: An in vitro study. Photochem. Photobiol..

[15] Ladiz M.A.R., Mirzaei A., Hendi S.S., Najafi-Vosough R., Hooshyarfard A., Gholami L. (2020). Effect of photobiomodulation with 810 and 940 nm diode lasers on human gingival fibroblasts. Dent. Med. Probl..

[16] Karoussis I.K., Kyriakidou K., Psarros C., Afouxenides P., Vrotsos I.A. (2021). Dosage Effects of an 810 nm Diode Laser on the Proliferation and Growth Factor Expression of Human Gingival Fibroblasts. J. Lasers Med. Sci..

[17] Etemadi A., Namin S.T., Hodjat M., Kosarieh E., Hakimiha N. (2020). Assessment of the Photobiomodulation Effect of a Blue Diode Laser on the Proliferation and Migration of Cultured Human Gingival Fibroblast Cells: A Preliminary In Vitro Study. J. Lasers Med. Sci..

[18] Etemadi A., Sadatmansouri S., Sodeif F., Jalalishirazi F., Chiniforush N. (2021). Photobiomodulation Effect of Different Diode Wavelengths on the Proliferation of Human Gingival Fibroblast Cells. Photochem. Photobiol..

[19] Ogita M., Tsuchida S., Aoki A., Satoh M., Kado S., Sawabe M., Nanbara H., Kobayashi H., Takeuchi Y., Mizutani K. (2015). Increased cell proliferation and differential protein expression induced by low-level Er:YAG laser irradiation in human gingival fibroblasts: Proteomic analysis. Lasers Med. Sci..

[20] Motlagh K.H., Azizi A., Ghadim N.M. (2023). In Vitro Effect of 445 nm Blue Laser and 660 nm Low-Level Laser on the Quantity and Quality of Human Gingival Fibroblasts. Photochem. Photobiol..

[21] Shingyochi Y., Kanazawa S., Tajima S., Tanaka R., Mizuno H., Tobita M. (2017). A Low-Level Carbon Dioxide Laser Promotes Fibroblast Proliferation and Migration through Activation of Akt, ERK, and JNK. PLoS ONE.

[22] Saygun I., Karacay S., Serdar M., Ural A.U., Sencimen M., Kurtis B. (2008). Effects of laser irradiation on the release of basic fibroblast growth factor (bFGF), insulin like growth factor-1 (IGF-1), and receptor of IGF-1 (IGFBP3) from gingival fibroblasts. Lasers Med. Sci..

[23] Sadatmansouri S., Agahikesheh B., Karimi M., Etemadi A., Saberi S. (2022). Effect of Different Energy Densities of 915 nm Low Power Laser on the Biological Behavior of Human Gingival Fibroblast Cells In Vitro. Photochem. Photobiol..

[24] Pourzarandian A., Watanabe H., Ruwanpura S.M.P.M., Aoki A., Ishikawa I. (2005). Effect of Low-Level Er:YAG Laser Irradiation on Cultured Human Gingival Fibroblasts. J. Periodontol..

[25] Skopin M.D., Molitor S.C. (2009). Effects of near-infrared laser exposure in a cellular model of wound healing. Photodermatol. Photoimmunol. Photomed..

[26] Illescas-Montes R., Melguizo-Rodríguez L., García-Martínez O., de Luna-Bertos E., Manzano-Moreno F.J., Ruiz C., Ramos-Torrecillas J. (2019). Human Fibroblast Gene Expression Modulation Using 940 NM Diode Laser. Sci. Rep..

[27] Karimi M.R., Abdollahi S., Etemadi A., Hakimiha N. (2024). Investigating the Effect of Photobiomodulation Therapy with Different Wavelengths of Diode Lasers on the Proliferation and Adhesion of Human Gingival Fibroblast Cells to a Collagen Membrane: An In Vitro Study. J. Lasers Med. Sci..

[28] Oyebode O.A., Houreld N.N. (2022). Photobiomodulation at 830 nm Stimulates Migration, Survival and Proliferation of Fibroblast Cells. Diabetes Metab. Syndr. Obes. Targets Ther..

[29] Hawkins D.H., Abrahamse H. (2006). The role of laser fluence in cell viability, proliferation, and membrane integrity of wounded human skin fibroblasts following helium-neon laser irradiation. Lasers Surg. Med..

[30] Wiegand C., Dirksen A., Tittelbach J. (2024). Treatment with a red-laser-based wound therapy device exerts positive effects in models of delayed keratinocyte and fibroblast wound healing. Photodermatol. Photoimmunol. Photomed..

[31] Moretti L., Stalfort J., Barker T.H., Abebayehu D. (2022). The interplay of fibroblasts, the extracellular matrix, and inflammation in scar formation. J. Biol. Chem..

[32] Almadani Y.H., Vorstenbosch J., Davison P.G., Murphy A.M. (2021). Wound Healing: A Comprehensive Review. Semin. Plast. Surg..

[33] Sang Y., Roest M., de Laat B., de Groot P.G., Huskens D. (2021). Interplay between platelets and coagulation. Blood Rev..

[34] Raziyeva K., Kim Y., Zharkinbekov Z., Kassymbek K., Jimi S., Saparov A. (2021). Immunology of Acute and Chronic Wound Healing. Biomolecules.

[35] Rodrigues M., Kosaric N., Bonham C.A., Gurtner G.C. (2019). Wound Healing: A Cellular Perspective. Physiol. Rev..

[36] Eming S.A., Martin P., Tomic-Canic M. (2014). Wound repair and regeneration: Mechanisms, signaling, and translation. Sci. Transl. Med..

[37] Landén N.X., Li D., Ståhle M. (2016). Transition from inflammation to proliferation: A critical step during wound healing. Cell. Mol. Life Sci..

[38] Xue M., Jackson C.J. (2015). Extracellular Matrix Reorganization During Wound Healing and Its Impact on Abnormal Scarring. Adv. Wound Care (New Rochelle).

[39] Kohale B.R., Agrawal A.A., Sope A.B., Pardeshi K.V., Raut C.P. (2015). Low-level Laser Therapy: A Literature Review. Int. J. Laser Dent..

[40] Jere S.W., Houreld N.N., Abrahamse H. (2020). Photobiomodulation and the expression of genes related to the JAK/STAT signalling pathway in wounded and diabetic wounded cells. J. Photochem. Photobiol. B Biol..

[41] Martu M.-A., Luchian I., Mares M., Solomon S., Ciurcanu O., Danila V., Rezus E., Foia L. (2023). The Effectiveness of Laser Applications and Photodynamic Therapy on Relevant Periodontal Pathogens (*Aggregatibacter actinomycetemcomitans*) Associated with Immunomodulating Anti-rheumatic Drugs. Bioengineering.

